# Exploring the Possibility of Cryopreservation of Feline and Canine Erythrocytes by Rapid Freezing with Penetrating and Non-Penetrating Cryoprotectants

**DOI:** 10.1371/journal.pone.0169689

**Published:** 2017-01-10

**Authors:** Denys Pogozhykh, Yuliya Pakhomova, Olga Pervushina, Nicola Hofmann, Birgit Glasmacher, Gennadiy Zhegunov

**Affiliations:** 1 Institute for Transfusion Medicine, Hannover Medical School, Carl-Neuberg-Strasse 1, Hannover, Germany; 2 Institute for Multiphase Processes, Leibniz Universitaet Hannover, Callinstrasse 36, Hannover, Germany; 3 Institute for Problems of Cryobiology and Cryomedicine of the National Academy of Sciences of Ukraine, Kharkov, Ukraine; 4 Kharkiv State Zooveterinary Academy, Mala Danylivka, Kharkiv Region, Ukraine; Justus Liebig Universitat Giessen, GERMANY

## Abstract

Efficient application of veterinary blood transfusion approaches for small companion animals requires readily available supply of the donor material. This can be achieved by developing of effective biobanking technologies allowing long-term storage of donor blood components via cryopreservation. Transfusion of an erythrocyte concentrate allows the successful correction of various hematological pathologies, severe bleeding, and etc. While in the past there were several approaches to cryopreserve red blood cells of dogs, to our knowledge there is virtually no data on cryopreservation of feline erythrocytes. In this paper, we performed a comprehensive parameter optimization for low temperature storage of RBCs of both species. Here, the efficiency of single-component and multicomponent cryoprotective media as well as necessary time of pre-incubation with penetrating and non-penetrating cryoprotectants prior to rapid freezing is analyzed. This study showed that glycerol was not sufficient for cryopreservation of red blood cells of the studied species under the investigated conditions. Application of 10% (v/v) ME_2_SO allowed for a significant reduction of canine and feline erythrocytes hemolysis after thawing. 17.5% hydroxyethyl starch demonstrated the highest cryoprotective activity for both species. It was found that dog RBCs should be incubated in cryoprotective media for 30 min at 22°C prior to freezing, while for cat RBCs 20 min is sufficient. Combination of CPAs was less effective. Presented data may be considered in further studies in veterinary transfusion and blood banking optimization.

## Introduction

Development of methods for low-temperature preservation and long-term storage of red blood cells (RBC) of domestic animals, particularly dogs and cats, is one of the actual problems in veterinary medicine [1,2,3]. The acute interest in this issue is determined by the growth of hematological diseases in these animals, low number of donor individuals, complexity of selection of blood according to phenotypic characteristics, as well as limited storage period at 4°C [4,5,6,7].

Transfusion of the erythrocyte concentrate may be also beneficial in the cases of severe blood loss, such as accidents, surgeries, and etc. [8]. The advantage of erythrocyte concentrate transfusions, in comparison with the whole blood, is the standardized amount of contained erythrocytes and hemoglobin along with minimal presence of citrate, leukocytes, platelets, and degradation products [8,9]. At the same time the total volume required for transfusion can be reduced.

In this work, we were interested on the comparison between the possibility for cryopreserving red blood cells of different animal species taking into consideration their peculiarities. Though canine and feline erythrocytes share many structural features of membranes and rates of biochemical reactions, these cells remain different in composition of protein and lipid membrane components [10,11] and peculiarities of homeostasis [12]. Besides, red blood cells of dogs and cats have a slight variation in size with an average volume of 41–51 μm^3^ and 62–74 μm^3^, respectively [13]. RBCs of the studied species exhibit different contents of sodium ions: 104 mmol/l is present in feline erythrocytes and 135 mmol/l in canine erythrocytes [14]. Moreover, the content of hemoglobin (90-170 g/l for cats and 120–170 g/l for dog) and its structure also differs among these species [15,16].

Therefore, in order to obtain high survival rates of thawed feline and canine erythrocytes, it is necessary to find the optimal conditions for cryopreservation for each of the studied species, i.e. select the most effective cryoprotectant (CPA) and find optimal concentration and time of pre-incubation with cells.

To our knowledge, there are virtually no studies on cryopreservation of erythrocytes of cats. As to cryopreservation of dog erythrocytes, several methods have been developed with application of ultra-low (-196°C) and moderately low (-80°C) temperatures with glycerol as a cryoprotectant [17]. These methods allow effective maintenance of frozen canine RBCs with only a minor damage after thawing. However, such approaches are not easily available for practical application in veterinary biobanking due to the complicated and time-consuming processes as well as due to glycerolization of the cells prior freezing and deglycerolization after thawing.

Several groups also studied efficiency of cryopreservation of canine RBCs with application of non-penetrating CPAs, such as PEO-1500 [18] and hydroxyethyl starch (HES) [19,20,21,22,23]. The advantages of cryopreservation methods with non-penetrating cryoprotectants for RBCs include the possibility to eliminate the washing steps prior to transfusion. However, the drawback of such CPAs is the presence of latent damage in thawed erythrocytes, which manifests itself in lowering the osmotic stability when they are placed in isotonic medium [24,25].

Lately, many researchers are actively engaged in the development of multicomponent cryoprotective media, containing CPAs of differing physical and chemical properties and mechanisms of the protective effect. Such approach resulted in successful freezing of rat mesenchymal stem cells [26], human platelets and erythrocytes [27,28], human liver hematopoietic cells [29], and certain plants [30]. Therefore, analysis of cryoprotective action of penetrating (glycerol, ME_2_SO) and non-penetrating (HES) CPAs, development of multicomponent media on their basis, as well as the selection of conditions of incubation of cells in cryoprotective solutions is the actual and highly promising direction for solving the problem of low-temperature preservation of RBCs of the companion animals.

The aim of this work was to evaluate the possibilities for cryopreservation of RBCs of dogs and cats with rapid cooling rates under various conditions of pre-treatment with CPAs.

## Materials and Methods

The objects of the study were erythrocytes isolated from the blood of healthy adult male mongrel dogs *(Canis familiaris)* and cats *(Felis catus)*. Both species were kept in the State Utility "Center of animal care" (Kharkov, Ukraine) and in the University of Veterinary Medicine Hannover, TiHo (Hannover, Germany). Manipulations with animals were carried out by professional veterinarians according to the international principles of the European Convention for the Protection of Vertebrate Animals used for Experimental and Other Scientific Purposes (ETS No. 123, 1986). All manipulations with animals in the University of Veterinary Medicine Hannover were carried out in strict accordance with German Animal Welfare Regulations and were approved by the Ethics Committee of Lower Saxony, Germany. All manipulations with the animals in the Kharkiv State Zooveterinary Academy were carried out in strict accordance with the Universal Declaration on Animal Welfare and National Animal Welfare Regulations and were approved by the Bioethics Committee of the Institute for Problems of Cryobiology and Cryomedicine of the National Academy of Sciences of Ukraine (Ethic votum No.3-1109-2013). The blood was retrieved out of brachial vein by venipuncture in accordance with the acting bioethical regulations. Dogs do not require anesthesia during the venipuncture blood retrieval. Cats were put under sedation with application of Sedazin (Biowet Puławy Ltd., Poland) with the acting substance Xylazine. Sedative analgesic was administered intramuscularly or subcutaneously at the rate of 3 mg / kg body weight. Xylazine action starts after 5 to 10 minutes after intramuscular injection. No animals were sacrificed in the frames of this study.

Dogs at the age from 3 to 5 years, weighing from 20 to 25 kg, and cats at the age from 1 to 5 years, weighing from 4.5 to 5 kg, were included in the experimental groups. The volume of whole blood obtained from one dog was 20 ml, and from one cat from 7 to 10 ml.

Preparation of blood was performed with citrate-glucose anticoagulant preservative and kept no more than 4 hours at 4°C prior to experimental procedures. Erythrocyte concentrate was obtained by centrifugation of the whole blood at 1500g for 5 min at 22°C, followed by removal of plasma and buffy coat. Afterwards, erythrocytes were washed three times in a 4-fold volume of isotonic saline solution (150 mM NaCl, 10 mM Tris-HCl, pH 7.4) by centrifugation at 1500g for 5 min at 22°C. RBC concentrates of dogs possessed on average 40% haematocrit level, while cat RBC concentrates usually had 35% haematocrit level. Therefore, blood samples with corresponding parameters for each species (40% for dogs and 35% for cats) were taken into experiment.

Stock solutions of CPAs were mixed with RBCs in 1:1 ratio (v/v). Durations of incubation of animal erythrocytes in the cryoprotective solutions were 10, 20 and 30 min at 22°C (room temperature). The samples were frozen in 5 mL cryo vials (Eppendorf, Hamburg, Germany) by immersion into liquid nitrogen (rapid freezing protocol). The cooling rate under such conditions is 2.5°C/sec [31]. Thawing of the samples was performed by transferring of container from the liquid nitrogen (-196°C) to a water bath with a constant shaking at 42°C. The samples were completely thawed in approximately 50–60 sec. Erythrocytes diluted 1:1 (v/v) in isotonic saline solution served as a control.

The following end concentrations of CPAs were used in cryopreservation media for freezing erythrocytes: ME_2_SO (5, 7.5, 10, 15, 20%); glycerol (5, 7.5, 10, 20%), HES 200 (6.25, 10, 17.5, 20%) as well as HES in combination with ME_2_SO or glycerol in various proportions presented in corresponding tables. CPAs, their certain concentrations and combinations were selected based on the range applied in the research laboratories for the cryopreservation of red blood cells of humans and certain domestic animals (dog, cattle, and horse) [18, 21].

Cryoprotective medium and washing solutions were prepared on 10 mM Tris-HCl (pH 7.4) buffer containing 150 mM NaCl. Washing the erythrocytes from cryoprotective media was carried out by serial centrifugation steps at 1500g. At the first step thawed erythrocytes were mixed in equal volume ratio with 0.6 M solution of NaCl, 10 mM Tris-HCl, pH 7.4, followed by two times centrifugation with 150 mM solution of NaCl, 10 mM Tris-HCl, pH 7.4 (1:1, v/v). In case of application of HES, the washing steps were excluded due to the extracellular nature of this CPA and lack of cytotoxicity.

Concentration of total hemoglobin in the cell suspension and the level of free hemoglobin in the supernatant were measured with CPK-3-01 photocolorimeter (ZOMZ, Russia) at a wavelength of 540 nm using Drabkin’s transforming solution (Diago-T, Russia) to convert hemoglobin into cyanmethemoglobin. The percentage of hemolyzed cells was calculated by the formula:

$$H_{\%}=\frac{{Hb}_{free}}{{Hb}_{total}}\times (1-Ht)\times 100\%.$$

Osmotic fragility was determined by the method [32], evaluating the stability of cells in hypotonic NaCl solutions ranging from 0.1% to 0.9%.

Statistical significance of differences between the studied groups was calculated with application of Mann-Whitney test. Results were considered statistically different with a significance levels of p <0.05.

## Results

### Hemolysis levels of erythrocytes after cryopreservation with single-component CPA solutions

#### Control group

Erythrocytes of cats and dogs from the control group were exposed in isotonic saline solutions for up to 30 min after preparation without any further impact factors. The data is presented as the level of hemolysis of RBCs in Table 1. Survival of erythrocytes of both species was higher than 99%.

**Table 1:** Control group. Level of hemolysis of erythrocytes of dogs and cats depending on the time of exposure in isotonic saline solutions (mean±SD, n = 5).

Sample	Hemolysis after 10 min incubation, [%]	Hemolysis after 20 min incubation, [%]	Hemolysis after 30 min incubation, [%]
Dog erythrocytes	0.39±0.31	0.59±0.70	0.90±0.91
Cat erythrocytes	0.30±0.21	0.78±0.17	0.94±0.60

Results of the influence of cryopreservation procedures on erythrocytes of cats and dogs with single-component CPA solutions are presented in Table 2. The data is presented as the level of hemolysis of RBCs after freezing-thawing-washing depending on the time of pre-incubation in CPA solutions.

**Table 2:** Level of hemolysis of erythrocytes of dogs and cats after freezing-thawing-washing depending on the time of exposure in single-component CPA solutions (mean±SD, n = 5).

CPA	End concentration of CPA in solution, [%]	Hemolysis after 30 min incubation with CPA without freezing, [%]	Hemolysis after freezing-thawing, [%]		
			Pre-incubation time with CPA, 10 min	Pre-incubation time with CPA, 20 min	Pre-incubation time with CPA, 30 min
Dog erythrocytes					
Glycerol	5	3.8±0.72	99.5±0.33	99.5±0.25	99.3±0.10
	7.5	5.2±0.93	99.7±0.40	99.4±0.20	99.1±0.15
	10	7.5±0.73	99.5±0.61	99.4±0.15	98.7±0.92
	20	9.0±0.95	99.6±0.30	93.2*±*0.89	75.4±1.05
ME_2_SO	5	0.11±0.2	99.5±0.31	99.4±0.21	98.7±1.01
	7.5	0.17±0.3	76.45±1.0	65.42±0.84	49.51±1.11
	10	0.21±0.5	60.1±1.32	42.41±1.06	26.3±1.12
	20	1.21±0.6	98.0±1.25	74.60±1.20	56.40±1.17
HES	6.25	0.17±0.7	75.4±1.20	70.20±1.29	58.72±1.55
	10	0.28±0.7	74.4±1.58	70.1±1.76	43.1±1.98
	17.5	0.55±0.5	34.76±1.86	30.1±3.02	16.10±1.15
	20	1.28±0.25	57.40±3.10	42.4±2.23	33.30±3.31
Cat erythrocytes					
Glycerol	5	0.85±0.04	99.41±0.20	99.40±0.12	99.30±0.15
	7.5	1.53±0.06	99.11±0.44	99.0±0.20	99.11±0.15
	10	3.22±0.06	97.0±0.31	84.24±2.24	90.11±1.82
	20	5.26±0.48	82.10±1.38	80.14±1.84	89.72±1.91
ME_2_SO	5	0.19±0.05	90.51±1.5	85.41±2.5	80.70±0.92
	7.5	0.40±0.07	86.9±1.89	52.21±1.62	69.30±1.89
	10	0.50±0.08	76.81±1.37	34.41±1.88	46.31±1.51
	20	2.0±0.16	96.47±1.17	67.40±1.35	80.51±1.51
HES	6.25	0.95 ±0.9	78.40±1.5	63.17±1.10	75.72±1.22
	10	2.85 ±1.15	75.0±1.26	62.36±1.75	79.54±1.82
	17.5	6.0±1.88	40.91±2.02	17.15±2.05	27.90±2.18
	20	22.4±2.70	56.80±2.40	37.41±2.54	43.36±2.60

### Erythrocytes of dogs

#### Glycerol

Hemolysis of RBCs after 30 min exposure with 5, 7.5, 10, and 20% glycerol solutions at 22°C increases insignificantly with the raise of concentrations in comparison to control (Table 2). Thus, studied incubation period and concentrations of glycerol are not significantly injurious for erythrocytes of dogs. However, freezing-thawing after 10, 20, or 30 min of exposure in 5, 7.5, and 10% solutions of glycerol resulted in complete red cell death (Table 2). Only the CPA solutions containing 20% glycerol combined with 30 min incubation allowed lowering the level of hemolysis to 75.4%. Thus, under the studied conditions, the application of glycerol as CPA appeared completely inefficient with a rapid freezing-thawing protocol.

#### ME_2_SO

Hemolysis of RBCs after 30 min exposure with 5, 7.5, 10, and 20% ME_2_SO solutions at 22°C increases insignificantly with the raise of concentrations in comparison to control (Table 2). Thus, ME_2_SO has no direct significant pathological effects on red blood cells of dogs within the studied concentrations and incubation period. However, cells pre-incubated for 10, 20, and 30 min in 5% solution of ME_2_SO, were subjected to virtually complete hemolysis after freezing-thawing. That is, such low concentrations of ME_2_SO did not provide cryoprotective activity. Increasing the final concentration of ME_2_SO in the cryoprotective solution to 7.5% resulted in significant lowering of hemolysis level. The minimal level of hemolysis (26.3%±1.12) was obtained with application of 10% ME_2_SO and 30 min pre-incubation. Further increasing of ME_2_SO concentration to 20% has proven to be less effective. This may be determined by the known cytotoxic effect of ME_2_SO in higher concentrations.

#### HES

Direct effect of HES on red blood cells of dogs was evaluated. After 30 min exposure with 6.25, 10, 17.5, and 20% HES solutions, hemolysis level of RBCs increased insignificantly in comparison to control. However, after 10, 20, and 30 min exposure in 6.25 and 10% solutions of HES and subsequent freeze-thawing, hemolysis of dog erythrocytes was rather significant (Table 2). Raising the concentration of HES to 17.5% resulted in significant lowering of RBC hemolysis after freezing-thawing. Yet, further increasing of the concentration of HES to 20% resulted in growth of the number of hemolyzed red blood cells after thawing. With increasing of pre-incubation time, the number of hemolytic cells was reduced in all studied HES solutions. The lowest level of hemolysis in thawed erythrocytes (16.10%±1.15) was obtained after pre-incubation for 30 min and subsequent cryopreservation in the 17.5% HES solution.

### Erythrocytes of cats

#### Glycerol

Hemolysis of RBCs after 30 min exposure with 5, 7.5, 10, and 20% glycerol solutions at 22°C increases insignificantly with the raise of concentrations in comparison to control (Table 2). Freezing-thawing of cat erythrocytes after 10, 20, or 30 min of exposure in 5, and 7.5% solutions of glycerol resulted in complete cell death (Table 2). 10% glycerol did not lead to significant reduction in hemolysis of erythrocytes after freezing-thawing procedures. Further increase in glycerol concentration to 20% allowed reducing hemolysis of thawed erythrocytes to the maximum with this CPA, but it was still insufficient to achieve satisfactory survival rates. Optimal pre-incubation time for glycerol for cat RBCs in the case was 20 min.

#### ME_2_SO

ME2_S_O also did not have direct damaging effect on the cat erythrocytes within the studied variations. Hemolysis of erythrocytes of cats, incubated in 5, 7.5, 10, and 20% solutions of ME_2_SO at 22°C for 30 min was insignificant (Table 2). Freezing-thawing of cat erythrocytes pre-incubated in 5% ME_2_SO solution for 10, 20, or 30 min, resulted in complete hemolysis. Hemolysis level decreased after freezing-thawing in 7.5% ME_2_SO solution, however, such survival was still unsatisfactory. Increasing the concentration of this CPA to 10% resulted in significant decrease of hemolysis levels for cat erythrocytes after freezing-thawing. The lowest hemolysis level of thawed RBCs (34.41%±1.88) was obtained with 10% ME_2_SO and 20 min pre-incubation. However, further enhancing of ME_2_SO concentration to 20% increases hemolysis of thawed cells again.

#### HES

It was shown that HES does not possess direct significant hemolytic effect on the RBCs of cats within the studied parameters. Hemolysis levels of feline erythrocytes incubated in 6.25, 10, 17.5, and 20% solutions of HES at 22°C for 30 min without freezing were low. It was found that cryoprotective activity of HES was manifested in all the studied concentration and pre-incubation periods. However, after freezing-thawing of feline RBCs with HES in 6.25 and 10% concentrations, hemolysis levels were not sufficiently low. Increasing the concentration of HES to 17.5% resulted in significant lowering of hemolysis level of thawed feline erythrocytes. Application of HES in 20% concentration increased the number of hemolyzed RBCs after thawing. Time of pre-incubation with HES also affected the survival of cat erythrocytes after freezing-thawing. According to the experimental data, 20 min was the most optimal pre-incubation time period for freezing feline RBCs with HES as a CPA. Thus, the highest survival of thawed cat erythrocytes was achieved after exposure for 20 min and subsequent cryopreservation in 17.5% HES solution.

### Hemolysis levels of erythrocytes after cryopreservation with combined CPA solutions

Results of the influence of cryopreservation procedures on erythrocytes of cats and dogs with combined CPA solutions after pre-incubation at 22°C are presented in Table 3. The data is presented as the level of hemolysis of RBCs after freezing-thawing-washing depending on the time of exposure in CPA solutions.

**Table 3:** Level of hemolysis of erythrocytes of dogs and cats after freezing-thawing-washing depending on the time of exposure in multicomponent CPA solutions (mean±SD, n = 5).

Composition of CPA solution	Hemolysis after 30 min incubation with CPA without freezing, [%]	Hemolysis after freezing-thawing, [%]		
		Pre-incubation time with CPA, 10 min	Pre-incubation time with CPA, 20 min	Pre-incubation time with CPA, 30 min
Dog erythrocytes				
5% Glycerol + 12.5% HES	1.85±0.13	99.7±0.54	95.10±3.1	87.9±1.91
7.5% Glycerol + 12.5% HES	3.46±0.29	99.30±0.33	99.0±0.52	78.30±1.87
15% Glycerol + 12.5% HES	39.50±2.27	99.60±0.21	70.0±1.90	59.01±2.09
5% ME_2_SO + 12.5% HES	6.01±2.26	99.50±0.10	99.51±0.47	89.0±1.08
7.5% ME_2_SO + 12.5% HES	12.14±3,2	99.31±0.31	99.10±0.21	78.9±1.05
Cat erythrocytes				
5% Glycerol + 12.5% HES	1.91±0.12	90.41±1.52	44.01±1.51	75.60±1.37
7.5% Glycerol + 12.5% HES	4.01±0.31	89.31±3.01	25.50±1.88	46.20±2.04
15% Glycerol + 12.5% HES	21.1±2.25	88.0±0.99	48.20±1.69	93.70±1.08
5% ME_2_SO + 12.5% HES	6.11±2.26	99.8±0.30	79.7±0.80	83.0±0.87
7.5% ME_2_SO + 12.5% HES	10.0±2.00	98.3±0.33	85.0±2.18	90.10±1.15

### Erythrocytes of dogs

#### Glycerol + HES

Cryopreservation of dog erythrocytes incubated in combined CPA solutions of glycerol + HES at a temperature of 22°C for 30 min without further freezing did not cause significant hemolysis of erythrocytes, except the concentrated solution of 15% glycerol + 12.5% HES, which showed a significant hemolytic effect (Table 3). Application of 5 as well as 7.5% glycerol in combination with 12.5% HES led to virtually complete hemolysis of dog erythrocytes after freezing-thawing, when the cells were pre-incubated for 10, 20, or 30 min. Increasing the concentration of glycerol to 15% after pre-incubation for 30 min led to a higher levels of survival of the cells after thawing, though still not sufficient. The highest survival level within the studied parameters for combination of glycerol and HES was only 59.01%±2.09.

#### ME_2_SO + HES

Cryopreservation of dog erythrocytes incubated in combined CPA solutions of ME_2_SO + HES at a temperature of 22°C for 30 min without further freezing also did not result in significant hemolysis (Table 3). However, freezing of RBCs of dogs in the presence of ME_2_SO in concentration of 5 and 7.5% in combination with 12.5% HES did not provide a satisfactory number of preserved cells under any of the studied conditions. Freezing, thawing, and washing procedures under these parameters did not provide more than 12% of live cells.

### Erythrocytes of cats

#### Glycerol + HES

Cryopreservation of cat erythrocytes incubated in combined CPA solutions of glycerol + HES at a temperature of 22°C for 30 min without further freezing did not cause significant hemolysis, except of the solution containing 15% glycerol + 12.5% HES, which possessed significant hemolytic effect (Table 3). Cryopreservation of cat erythrocytes with combined CPAs glycerol + HES significantly increased survival after thawing. Application of 5% glycerol in combination with 12.5% HES, after pre-incubation for 20 min at 22°C, allowed reducing hemolysis level to 44.01%±1.51. A higher level of survival (hemolysis level 25.50%±1.88) was obtained after exposure of the erythrocytes for 20 min in 7.5% glycerol and 12.5% HES.

#### ME_2_SO + HES

Cryopreservation of cat erythrocytes incubated in combined CPA solutions of ME_2_SO + HES at a temperature of 22°C for 30 min without further freezing did not cause significant RBC hemolysis (Table 3). However, application of the combined CPA solutions containing ME_2_SO + HES was not effective for cryopreservation of erythrocytes of cats. Freezing-thawing-washing procedures with either 5 or 7.5% ME_2_SO in combination with 12.5% HES resulted in virtually complete hemolysis of cells, regardless of pre-exposure time.

### Osmotic fragility of erythrocytes after cryopreservation procedures

Osmotic fragility is among the main indicators associated with RBC membrane abnormalities. Based on the analysis of hemolysis levels after freezing-thawing procedures, combined and single component CPA solutions, which allowed achieving highest survival rates of RBCs, were selected for the further studies on the effect of osmotic resistance of erythrocytes after cryopreservation.

### Erythrocytes of dogs

**Fig 1:**
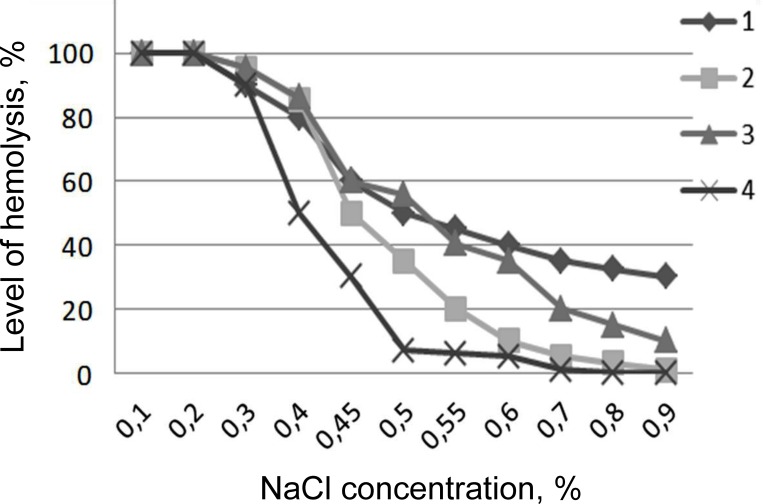
Osmotic fragility of canine erythrocytes after cryopreservation procedures. 1. Cells cryopreserved under the protection of 17.5% HES; 2. Cells cryopreserved under the protection of 7.5% DMSO + 12.5% HES; 3. Cells cryopreserved under the protection of 7.5% glycerol + 12.5% HES; 4. Control cells.

Osmotic fragility of thawed erythrocytes of dogs was higher in comparison to control in all the experimental samples (Fig 1). The highest instability of erythrocyte membranes was observed after freezing-thawing with 17.5% of HES. This indicates the presence of latent damage at cryopreservation of canine RBCs with this CPA, which is manifested immediately after the transfer of red cells in isotonic NaCl medium. Adding a penetrating cryoprotectant to solution with HES, especially ME_2_SO, reduces the instability of erythrocyte membranes (Fig 1), although does not improve the survival rate after freezing-thawing (Tables 2 and 3).

With a decrease in the concentration of NaCl osmotic instability of dog erythrocytes gradually increased. Cell hemolysis in the control group was approximately 30% at 0.45% NaCl concentration. At the same time, hemolysis of RBCs after freezing-thawing with cryoprotective solutions containing 17.5% HES, as well as combination of 7.5% glycerol + 12.5% HES was significantly higher and reached up to 60%. The lowest levels of osmotic fragility were observed in erythrocytes frozen in a solution containing 7.5% ME_2_SO + 12.5% HES.

### Erythrocytes of cats

**Fig 2:**
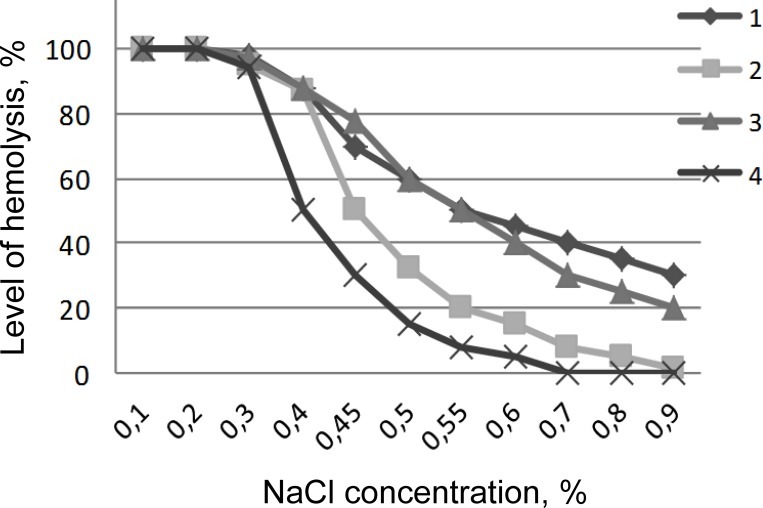
Osmotic fragility of feline erythrocytes after cryopreservation procedures. 1. Cells cryopreserved under the protection of 17.5% HES; 2. Cells cryopreserved under the protection of 7.5% DMSO + 12.5% HES; 3. Cells cryopreserved under the protection of 7.5% glycerol + 12.5% HES; 4. Control cells.

The tendency in parameters of osmotic fragility of thawed erythrocytes of cats was similar to that in canine samples (Fig 2). The highest instability of erythrocyte membranes was likewise observed after freezing-thawing with 17.5% of HES. It also indicates the presence of latent damage after cryopreservation with this CPA, which is manifested immediately after the transfer of red blood cells in isotonic NaCl solutions. Adding penetrating cryoprotectants to the HES solution, in particular ME_2_SO, reduces the level of instability of erythrocyte membranes (Fig 2), although it does not improve the survival level after freezing-thawing (Tables 2 and 3).

With a decrease in the concentration of NaCl osmotic instability of feline RBCs increased. Hemolysis of cells from the control group was approximately 30% at 0.45% NaCl concentration. However, hemolysis of erythrocytes frozen in cryoprotective solutions containing 17.5% HES as well as combination of 7.5% glycerol + 12.5% HES was more than 2 fold higher compared to the control. Therefore, the presence of latent damage, which may not immediately manifest itself after thawing, should be highly considered. The lowest levels of osmotic fragility in 0.45% NaCl were observed in feline erythrocytes frozen in a solution containing 7.5% ME_2_SO + 12.5% HES.

## Discussion

The optimal choice for combination of conditions towards cryopreservation is a crucial factor to ensure high survival rates of red blood cells. When selecting conditions for cryopreservation of feline and canine erythrocytes, it is first of all necessary to select a cryoprotectant with minimal cytotoxicity and at the same time high cryoprotective activity. Besides, it is important to optimise concentration of such CPA and duration of pre-incubation of the cells in the cryoprotective solution. In this study, three substances were analysed for cryoprotective properties: glycerol, ME_2_SO and HES, as well as solutions based on their combinations. It should be specifically noted that, to our knowledge, none of these chemical compounds has been studied so far for cryoprotective activity for erythrocytes of cats.

Glycerol in the concentration of 40% was previously investigated for its cryoprotective activity during freezing of dog erythrocytes. [17] Furthermore, two methods of cryopreservation, such as at ultra-low (-196°C) and at moderately low (-80°C) temperatures, were developed. Both methods demonstrated to provide sufficient high level of viable thawed RBCs. However, the main drawback of these methods is a long and laborious procedure of introducing this CPA to the cell suspension, which includes a four-step process. Such method of cryoprotectant introduction probably serves to reduce the cytotoxic effect of the high glycerol concentrations.

In our study we applied a single-step of glycerol introduction followed by pre-incubation of the cells with a CPA solution prior freezing. This could be the main reason for the difference in our results in comparison to those previously cited [17]. Apparently this single-step method of introduction of this CPA is not entirely optimal for the RBCs of our analysed species, since the studied 10 and 20% glycerol solutions show high osmotic activity and may cause hypertonic stress in feline and canine erythrocytes. It is known that a major damaging factor of hypertonic stress is the excessive cell dehydration, and as a consequence, a critical change in its volume [33].

At the same time, other studies showed that glycerol provides protection to human erythrocytes from hypertonic stress caused by the action of other substances [34]. Such protective effect of glycerol is associated with its ability to quickly permeate through the membrane of human erythrocytes (0.38 ± 0.08 C × 10^−7^, m/sec) [35], and thereby hindering the concentration of its intracellular composition [34]. Furthermore, glycerol prevents significant changes in the volume of erythrocytes placed into hypertonic medium. However, such protective mechanism of action against the damaging effects of hypertonic stress most likely cannot be realized in dog RBCs. This is determined by peculiarities of the membranes of canine erythrocytes, which have low permeability to glycerol (0.046 ± 0.09 C × 10^−7^, m/sec) [18]. One can assume that if this CPA does not have sufficient time to evenly distribute between the extra- and intracellular environment, it becomes a factor that triggers damage to the dog's red blood cells, not only during exposure time, but also in the freezing-thawing process. This results in a complete or partial cell death, which is confirmed by our data. Information on glycerol permeability through the cell membranes of RBCs of cats is absent in the literature. Therefore, it is difficult to speculate if the damaging effect of glycerol on feline erythrocytes is related to the osmotic changes. Application of 7.5 and 5% glycerol solutions also proved to be ineffective showing no sufficient cryoprotective activity during freezing of erythrocytes of dogs and cats in such concentrations.

Analysis of cryoprotective action of ME_2_SO at freezing of canine and feline erythrocytes revealed certain advantages of this compound over glycerol, despite the fact that it requires prolonged exposure of cells in a CPA solution to provide the maximal cryoprotective effect. Advantage of ME_2_SO reveals significantly higher survival rates of erythrocytes of dogs and cats after freezing-thawing processes in comparison to glycerol. In our studies maximal survival rates of erythrocytes required 10% concentration of ME_2_SO. It is known that the permeability coefficient for ME_2_SO through membranes of canine erythrocytes is significantly higher (0.57 ± 0.12 C × 10^−7^, m/sec) in comparison to glycerol. [18] Therefore, the required time for distribution of this CPA between extra- and intracellular environment should be much shorter. This may explain the fact that in our data ME_2_SO demonstrate a cryoprotective effect already after 10 min exposure. Additionally, a more rapid penetration of ME_2_SO into RBCs reduces the chance of hypertonic stress damage. Higher rates of canine and feline RBC survival after prolonged exposure to ME_2_SO solution may be attributed to the maximum amount of CPA entering erythrocytes, thus implementing cryoprotective action at a higher extent. Similar effect was described at freezing human erythrocytes using dextran and glucose in combination with disaccharides [31]. Authors showed that enhancing of resistance of erythrocytes to cryopreservation was achieved by maximizing the intracellular accumulation of trehalose. Application of 5 and 7.5% ME_2_SO was less effective, since this CPA in such concentrations did not exhibit sufficient cryoprotective activity during freezing of canine and feline erythrocytes. At the same time, in our studies, increasing the concentration to 20% reduced survival rates of RBCs, which may be explained by cytotoxic action of this cryoprotectant. It was shown that 10-20 molar concentrations of ME_2_SO promote formation of transient aqueous pores in the lipid bilayer due to the interaction with acyl chains of phospholipids. These pores contribute to the damage of asymmetric lipid bilayer [36].

In our work we have also studied the effects of non-penetrating cryoprotectant HES, which is considered to be a non-cytotoxic compound which does not require removal from the RBC suspension prior to transfusion [19,20,22,23]. Our studies have shown that 17.5% was the most effective concentration of HES for expressing the cryoprotective effect during freezing of canine and feline erythrocytes. However, our data does not completely correspond to the findings of other authors, who showed the highest survival rates for RBCs of dogs frozen at 12.5% HES solution [21]. Such discrepancy can be explained by the differences in preparation of solutions as well as diversity in grades and sources of this CPA.

It is believed that the mechanism of action of non-penetrating cryoprotectants lies in their ability to adsorb on the cellular membrane and bind extracellular water thus inhibiting extra- and intracellular ice crystal formation [37]. The ability of non-penetrating CPAs to bind water depends on their concentration: the higher it is—the larger amount of water molecules can be bound with one molecule of cryoprotectant [38]. Perhaps this is one of the factors causing increase of survival rates of canine and feline RBCs with the application of higher concentration of HES.

However, the value of osmotic pressure of non-penetrating CPA solution, which increases with the raise of concentration, plays a crucial role in the outcome of cryopreservation of biological objects. Perhaps this can explain the drastic increase in erythrocyte hemolysis after cryopreservation with 20% HES.

It is known that non-penetrating cryoprotectants may interact with the surface of the cell membrane [39,40]. Hydrocarbon radicals in the molecular structure of non-penetrating CPAs allows them to participate not only in the hydrophilic, but also in hydrophobic interactions. It was shown that the highest survival of human RBCs cryopreserved with non-penetrative PEO-1500 requires 40 min incubation of the cells in the CPA solution [41]. It is believed that the action of PEO-1500 results in modifications in cytoskeleton-membrane complex of erythrocytes, which increases their resistance to cryopreservation [39]. We assume that improvements in stabilization of erythrocyte membranes of dogs and cats under the influence of HES may also require longer exposure to cryoprotective solution. At the same time, it should be noted that erythrocytes of cats need shorter period of exposure to obtain the highest levels of survival in comparison to dog RBCs. One can speculate that feline erythrocytes require less time to establish cell-cryoprotectant osmotic equilibrium.

The presented data suggests convenience of application of non-penetrating cryoprotectant HES in 17.5% concentration as a main component of the cryoprotective medium for freezing RBCs with a process which does not require a washing step after thawing.

However, high levels of osmotic fragility of studied erythrocytes after freezing-thawing with HES should be considered. Figs 1 and 2 show haemolysis of thawed RBCs in 0.45% NaCl solution exceeds 50%, which may indicate for the latent damage in the cell membranes.

In our studies, we have also performed modifications of CPA solution on the basis of HES by including penetrating CPAs in the media. Raising the osmotic stability of cryopreserved red cells is considered as one of the main criteria of efficiency of combined cryoprotective media, since it is one of the markers of membrane deterioration after the action of extreme factors in the process of cryopreservation.

Data on the application of cryoprotective media containing combinations of HES with glycerol and DMSO in different concentrations revealed varying levels of success in the preservation of feline and canine erythrocytes after freezing-thawing processes. Multidirectional character of changes of survival indicators in cryopreserved cells, as determined by the composition of CPA medium and the exposure time, was clearly observed. For example, introduction of glycerol or ME_2_SO to the CPA composition with 12.5% HES resulted in lowering RBC survival, as evaluated by detecting hemolysis levels, in comparison to the single-component CPA solution containing only HES. At the same time, application of glycerol in composition of cryoprotective medium based on 12.5% HES was more effective in comparison to combination with ME_2_SO for freezing of cat RBCs. For dog erythrocytes the differences between glycerol and ME_2_SO in combination with HES were not significant.

While the use of combined CPA solutions does not improve survival rates, it improves the osmotic resistance of thawed RBCs of the studied species. Application of 7.5% ME_2_SO in combination with 12.5% HES, contributed to the reduction of osmotic fragility of thawed erythrocytes in comparison to single-component 17.5% HES solution. Probably, ME_2_SO has a property to effectively reduce the osmotic fragility of RBCs when combined with non-penetrating CPAs. This assumption may be confirmed by the findings that combination of 5% ME_2_SO with 20% PEO (Mw 1500 and 2000) or Dextran (Mw 3500 and 10000) allows significantly lowering the levels of osmotic fragility of thawed erythrocytes [42]. Cryopreservation of RBCs under the protection of pluronic polyols in combination with ME_2_SO also allowed achieving high levels of survival [43]. In accordance to our previous studies on cryopreservation of human erythrocytes with OEGn = 25 as a CPA, we demonstrated that introduction of penetrating cryoprotectant dimethylacetamide into cryopreservation media has an effect of reducing the osmotic fragility [28]. Thus, it was found that 17.5% HES is efficient in protecting the erythrocytes of dogs and cats during cryopreservation, but such cells have latent defects. At the same time, application of combined solutions, containing HES and penetrating CPAs, reduces the latent damage of red blood cells after thawing.

## Conclusions

To summarize the data it is convenient to make the following overall conclusions:

1. It was found that all studied CPAs and cryoprotective media, except 15% Glyserol + 12.5% HES, do not exhibit pronounced damaging hemolitic effects on RBCs of dogs and cats prior to freezing during pre-incubation of up to 30 minutes.
2. Glycerol was ineffective for cryopreservation of RBCs of the studied animals at the studied conditions.
3. ME_2_SO possessed cryoprotective activity, but survival rates were still insufficient.
4. HES possessed the highest cryoprotective effect on the RBCs. HES was the most effective at 17.5% concentration, requiring 30 min of pre-incubation for the RBCs of dogs and 20 min for the RBCs of cats as the optimal condition for cryopreservation. At the same time erythrocytes thawed after cryopreservation with 17.5% HES possessed enhanced osmotic fragility.
5. Combination of penetrating and non-penetrating CPAs was less effective for RBCs survival in comparison to single-component CPA solutions, based on hemolysis levels after thawing.
6. At the same time, introduction of penetrating CPAs to cryoprotective media on the basis of 12.5% HES contributes to lowering the osmotic fragility levels of feline and canine erythrocytes after cryopreservation.

knowledge here obtained may be valuable for the studies in transfusion veterinary as well as for the optimization and standardization of blood banking procedures.
